# Effect of Chlorination on Microbiological Quality of Effluent of a Full-Scale Wastewater Treatment Plant

**DOI:** 10.3390/life11010068

**Published:** 2021-01-19

**Authors:** Ioanna Zerva, Nikolaos Remmas, Ifigeneia Kagalou, Paraschos Melidis, Marina Ariantsi, Georgios Sylaios, Spyridon Ntougias

**Affiliations:** 1 Department of Environmental Engineering, Laboratory of Wastewater Management and Treatment Technologies, Democritus University of Thrace, Vas. Sofias 12, 67132 Xanthi, Greece; izerva@env.duth.gr (I.Z.); nremmas@env.duth.gr (N.R.); pmelidis@env.duth.gr (P.M.); mariaria@env.duth.gr (M.A.); 2 Department of Civil Engineering, Democritus University of Thrace, Vas. Sofias 12, 67132 Xanthi, Greece; ikagkalo@civil.duth.gr; 3 Department of Environmental Engineering, Laboratory of Ecological Engineering and Technology, Democritus University of Thrace, Vas. Sofias 12, 67132 Xanthi, Greece; gsylaios@env.duth.gr

**Keywords:** waterborne pathogens, effluent quality, toxic cyanobacteria, chlorination, filamentous bacteria, microbial ecology of effluent

## Abstract

The evaluation of effluent wastewater quality mainly relies on the assessment of conventional bacterial indicators, such as fecal coliforms and enterococci; however, little is known about opportunistic pathogens, which can resist chlorination and may be transmitted in aquatic environments. In contrast to conventional microbiological methods, high-throughput molecular techniques can provide an accurate evaluation of effluent quality, although a limited number of studies have been performed in this direction. In this work, high-throughput amplicon sequencing was employed to assess the effectiveness of chlorination as a disinfection method for secondary effluents. Common inhabitants of the intestinal tract, such as *Bacteroides*, *Arcobacter* and *Clostridium*, and activated sludge denitrifiers capable of forming biofilms, such as *Acidovorax*, *Pseudomonas* and *Thauera*, were identified in the chlorinated effluent. *Chloroflexi* with dechlorination capability and the bacteria involved in enhanced biological phosphorus removal, i.e., *Candidatus* Accumulibacter and *Candidatus* Competibacter, were also found to resist chlorination. No detection of *Escherichia* indicates the lack of fecal coliform contamination. *Mycobacterium* spp. were absent in the chlorinated effluent, whereas toxin-producing cyanobacteria of the genera *Anabaena* and *Microcystis* were identified in low abundances. Chlorination significantly affected the filamentous bacteria *Nocardioides* and *Gordonia*, whereas *Zoogloea* proliferated in the disinfected effluent. Moreover, perchlorate/chlorate- and organochlorine-reducing bacteria resisted chlorination.

## 1. Introduction

Wastewater treatment plants (WWTPs) usually focus on the removal of nutrients and suspended solids in order to safely dispose of the effluent in a recipient aquifer. However, WWTPs could potentially constitute a source of the dispersal of microorganisms in the environment, as microbes are not completely removed during treatment [1,2]. Thus, the conventionally treated effluent may still include a pathogenic charge, such as bacteria and protozoa, including harmful cyanobacteria capable of producing toxins under certain conditions [2,3,4]. Moreover, chemical contaminants, such as pharmaceuticals and personal care products (PPCPs), are also released into the environment, along with the dispersal of antibiotic resistance genes, thus strengthening environmental concerns [4,5]. This is mainly due to the limited efficiency of the conventional treatment processes, a fact that can lead to increased human health risk associated with food web and water contamination [1,5,6,7]. As a consequence, the implementation of effective disinfection methods has become increasingly necessary in order to eliminate the potential microbial risk posed by the effluent discharge [6,8].

Several disinfection methods, such as chlorination [8,9], UV radiation [1,6,9,10], ozonation [6,11], as well as peracetic acid [12,13] and hydrogen peroxide addition [10,14,15,16], have been employed to improve microbiological effluent quality. Each disinfection method relies on a specific inactivation mechanism, such as DNA damage, enzyme denaturation, and cell wall and membrane disruption [6]. For instance, sodium hypochlorite (NaClO) exerts a strong oxidizing capacity, affecting cell membrane structure, protein functionality and DNA replication [17]. Moreover, advanced oxidation processes (AOPs), in which highly reactive hydroxyl radicals are produced by primary oxidants, such as ozone, hydrogen peroxide and oxygen in combination with UV or a catalyst, are suitable methods for the microbial inactivation of secondary treated wastewater [18].

Even though chlorination is a commonly applied disinfection method, its effect on the bacterial community’s structure in the effluent deserves further evaluation [8]. The microbiological quality of the effluent nowadays relies on the assessment of certain microbial indicators, which include total and fecal coliforms and enterococci. At the same time, little is known about the fate of waterborne pathogens able to resist disinfection, being released in aquatic environments [4,6,19]. In addition, coliform indicators may be susceptible to conventional chemical disinfectants, and possibly do not reflect precisely the occurrence of pathogens [19,20]. Indeed, the application of culture-dependent approaches provides a coarse estimation of the microbial risk in the discharged effluent, a fact that leads to an increased uncertainty regarding the evaluation of its microbiological quality [19]. On the other hand, massive amounts of sequencing data are obtained by the application of high-throughput molecular techniques, providing an accurate mapping of microbial communities, thus allowing the more precise evaluation of the disinfection methods applied in wastewater treatment [21].

Despite the fact that the effectiveness of common disinfection methods has been examined in various research works using microbial indicators [17,20,22,23], a few studies have been performed through the use of culture-independent techniques to access microbiological effluent quality [3,24]. The aim of the present study was to evaluate the effectiveness of chlorination as a disinfection method against bacterial pathogens and key physiological groups of bacteria detected in the effluent of a full-scale wastewater treatment plant. In order to achieve this goal, we investigated the bacterial communities in the effluent prior to and after chlorination using the high-throughput amplicon sequencing approach.

## 2. Materials and Methods

The effluent of a full-scale municipal wastewater treatment plant (Evros region, Thrace, Greece) consisting of a primary sedimentation tank, an aerobic tank and a secondary clarifier was analyzed to investigate the effect of chlorination on the microbiological quality of the effluent. The chlorination of the effluent in the wastewater treatment plant was performed by adding sodium hypochlorite to achieve a final residual chlorine concentration of 0.2 mg/L under a contact time of 30 min. Three distinct sampling procedures were made in a period of one month in order to collect altogether three non-chlorinated and three chlorinated effluent samples. All samples were collected in sterile containers and transferred at 5 °C within an hour to the laboratory for the immediate processing of the physicochemical analyses and DNA extraction. Genomic DNA was stored at −80 °C for downstream analyses.

### 2.1. Determination of Wastewater Physicochemical Characteristics

To determine the physicochemical characteristics, samples were collected from the influent (raw wastewater) and the effluent (treated wastewater discharged after chlorination in the recipient) of a full-scale wastewater treatment plant. Biochemical oxygen demand (BOD_5_), chemical oxygen demand (COD) and total suspended solids (TSS) were determined based on the protocols described in the standard methods for the examination of water and wastewater [25]. The cadmium reduction method was applied for the quantification of nitrate’s reduction to nitrite, whereas nitrites were assessed colorimetrically at 453 nm through the addition of sulfanilamide/(1-naphthyl)ethylenediamine-dihydrochloride indicator [25].

### 2.2. DNA Extraction, Illumina Sequencing, Diversity Indices Estimation and Statistical Analyses

Prior to and after chlorination, genomic DNA was obtained after effluent filtration and DNA extraction from the filter retaining the microorganisms (pore size of 0.22 μm) through the use of Wizard^®^ Genomic DNA Purification Kit (Promega, Madison, WI, USA), following the manufacturer’s instructions.

PCR amplification of the V1-V3 region of the 16S rRNA gene was conducted using the bacterial universal primers 27F (5′-AGR GTT TGA TCM TGG CTC AG-3′) and 519R (5′-GTN TTA CNG CGG CKG CTG-3′) [26]. A thermo-cycle program consisting of 3 min DNA denaturation at 94 °C, a pool of 30 cycles of 30 s DNA denaturation at 94 °C, 40 s primer hybridization at 53 °C, and 1 min DNA extension at 72 °C followed by a 5 min termination step at 72 °C, was carried out. The reaction mixture was prepared by using “Qiagen HotStarTaq Plus Master Mix Kit” (Qiagen, Valencia, CA, USA) and amplified products were purified via the use of Ampure XP beads (Pacific Biosciences, Menlo Park, CA, USA). Illumina sequencing reactions were conducted in a MiSeq apparatus at “Mr DNA”. Sequencing data were deposited in the SRA database at NCBI under the BioProject ID PRJNA681028.

All amplicons obtained were subjected to demultiplexing and trimming, and Ν(s)-containing sequences, or reads with inappropriate length size or low-quality, were discarded [27]. Assembled sequences were improved in quality and chimeras were excluded through the use of USEARCH v.11 [28]. The clustering of non-chimeric reads into operational taxonomic units (OTUs) (number of reads ≥ 2) was conducted by using the -cluster_otus option. Genus-level taxonomic assignments were made using RDP (Ribosomal Database Project). The MicrobiomeAnalyst platform was employed to calculate the diversity indices of the microbial communities of wastewater prior to and after chlorination [29]. Chao1, Fisher, Shannon and Simpson indices were assessed to uncover alpha-diversity. In order to access beta-diversity, principal coordinates analysis (PCoA) was conducted in the MicrobiomeAnalyst platform by using the Bray–Curtis algorithm, followed by permutational multivariate analysis of variance (PERMANOVA).

Analysis of variance (ANOVA) was performed using PAST v.3.25 [30] to assess differences among the relative abundances of the identified bacterial taxa, either prior to (marked as lowercase letters) or after chlorination (marked as uppercase letters). Student’s *t*-test was carried out to identify statistically significant differences for the calculated diversity indices prior to and after chlorination, at a probability p lower than 0.01 (**) or 0.05 (*).

## 3. Results

The BOD and COD removal efficiencies in the full-scale wastewater treatment plant exceeded 89% (Table 1). Moreover, the TSS were highly decreased in the effluent, indicating biosolids removal of 93.8 ± 0.2% (Table 1).

After quality filtering, 522,025 sequencing reads (out of 766,896 in total) were analyzed. The clustering of non-chimeric reads into operational taxonomic units (OTUs) resulted in the identification of 560 and 467 OTUs prior to and after chlorination, respectively.

**Figure 1:**
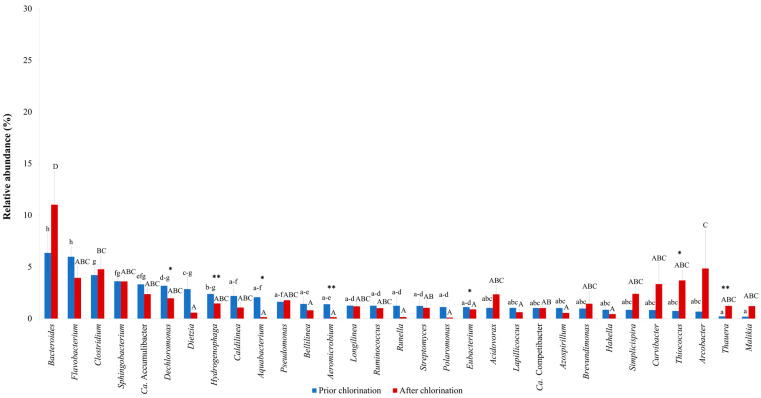
Major bacterial taxa in the effluent of a full-scale WWTP prior to and after chlorination. Letter(s) in common denote no statistically significant differences. (**) or (*) within the same taxon indicates statistically significant difference prior to and after chlorination, at *p* < 0.01 or *p* < 0.05, respectively.

The major bacterial taxa found in the effluent prior to and after chlorination are shown in Figure 1. Minor taxa (<1% of the total reads) are listed in Supplementary Table S1. The genus *Bacteroides* was a predominant taxon in the effluent prior to chlorination with a relative abundance of 6.36 ± 1.87% of the total reads, followed by members of the genera *Flavobacterium* (5.98 ± 0.99%), *Clostridium* (4.20 ± 0.86%), *Sphingobacterium* (3.61 ± 0.31%), *Candidatus* Accumulibacter (3.31 ± 0.29%) and *Dechloromonas* (3.17 ± 0.21%) (Figure 1). After disinfection, *Bacteroides* remained the dominant taxon, indicating the resistance of this genus to chlorination (Figure 1). In addition, *Arcobacter*, *Clostridium* and *Thiococcus* were among the major taxa detected after treatment with hypochlorite, representing 13.30 ± 5.30% of the total reads (Figure 1). Moreover, known denitrifiers from the genera *Acidovorax*, *Pseudomonas* and *Thauera*, and members of the intestinal tract, *Bacteroides*, *Clostridium* and *Ruminococcus*, were identified in the chlorinated effluent. *Chloroflexi* with dechlorination capability, i.e., *Caldilinea*, *Bellilinea* and *Longilinea*, as well as microorganisms involved in enhanced biological phosphorus removal, such as *Candidatus* Accumulibacter and *Candidatus* Competibacter spp., were also among the most abundant chlorination-resistant genera. On the other hand, *Aeromicrobium*, *Aquabacterium* and *Hydrogenophaga* were the most hypochlorite-susceptible genera (*p* < 0.01), followed by *Dechloromonas* and *Eubacterium* (*p* < 0.05). By contrast, the relative abundance of *Thiococcus* and *Thauera* increased after chlorination (Figure 1), indicating their effective adaptation to such kinds of water disinfection. In addition, the relative abundances of *Bacteroides* and *Acidovorax* were increased, but in a non-significant manner.

**Figure 2:**
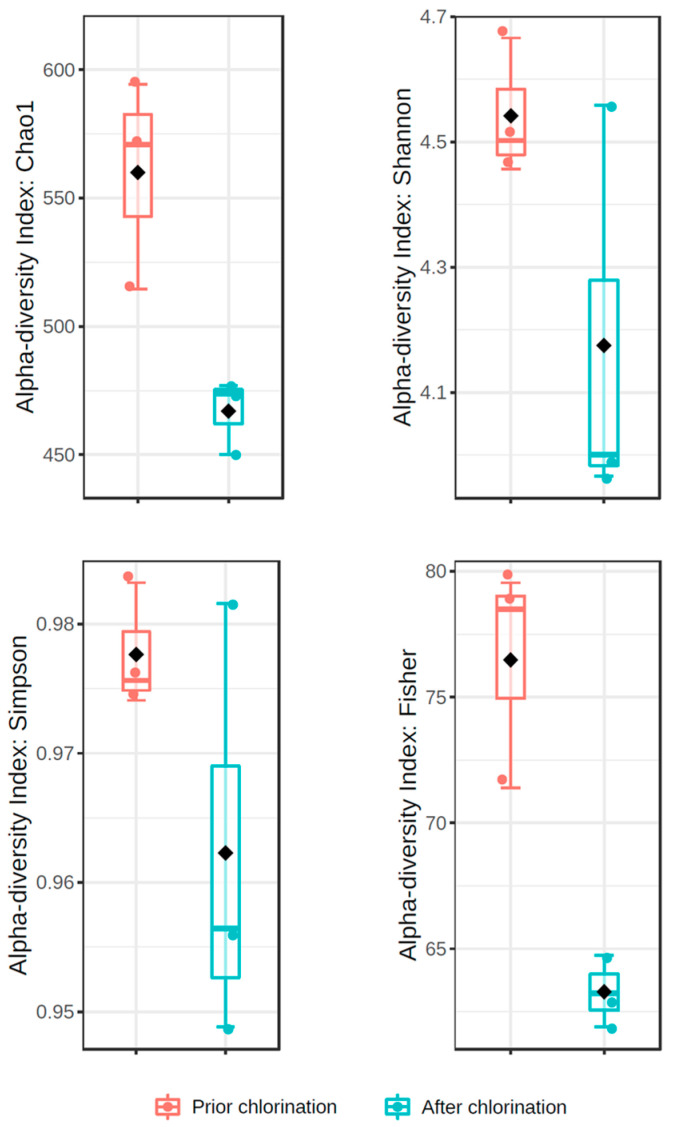
Diversity indices of bacterial communities in the effluent of WWTP prior to and after chlorination.

**Figure 3:**
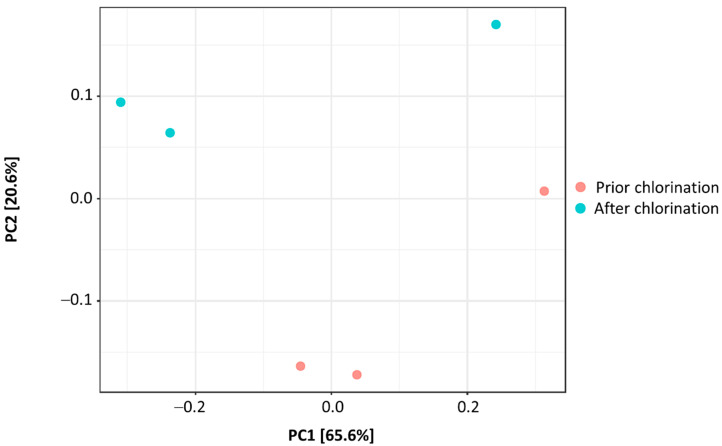
PCoA biplot showing beta diversity of bacterial communities in the effluent of WWTP prior to and after chlorination.

Bacterial richness was significantly decreased by chlorination (*p* < 0.05), resulting in the decrease in Chao1 index from 560 ± 24 to 467 ± 8 after disinfection (Figure 2). The Shannon diversity index was not statistically different in non-chlorinated and chlorinated effluent (4.54 ± 0.06 vs. 4.18 ± 0.19, respectively) (Figure 2). In addition, Simpson diversity did not significantly differ in the effluents prior to and after chlorination (0.98 ± 0.01 vs. 0.96 ± 0.01, respectively) (Figure 2), reaching values close to 1. On the other hand, Fisher’s diversity index lowered significantly from 76.47 ± 2.56 prior to chlorination to 63.27 ± 0.83 after chlorination (*p* < 0.01) (Figure 2). Pielou’s evenness indexes non-significantly differed in the effluent prior to and after chlorination (0.71 ± 0.02 and 0.67 ± 0.03, respectively). PCoA showed that the beta diversity of the bacterial communities did not statistically differ in the effluent prior to and after chlorination (*F*-value: 1.6101; R^2^: 0.287; *p* < 0.30) (Figure 3).

Perchlorate/chlorate-reducing bacteria [31], including *Arcobacter* spp. [32], together with organochlorine-reducing bacteria (Table 2) represented a high proportion of the total reads, i.e., 9.13 ± 1.26% and 10.13 ± 3.11%, in the effluent prior to and after chlorination, respectively.

Ammonia-oxidizing bacteria of the genera *Nitrosovibrio* and *Nitrosomonas*, and nitrite-oxidizing bacteria of the genera *Nitrospira* and *Candidatus* Nitrotoga, were the main nitrifying taxa identified (Table 3), with the relative abundance of *Nitrosovibrio* being statistically decreased by the addition of disinfectant. As expected, anammox bacteria of the genera *Candidatus* Kuenenia and *Candidatus* Anammoximicrobium were restricted in abundance (Table 4). Besides this, polyphosphate-accumulating organisms (PAOs) and their antagonistic glycogen-accumulating organisms (GAOs), which are involved in the enhanced biological phosphorus removal (EBPR) process, were resistant to chlorination since no statistically significant differences were detected in their relative abundances (Table 4).

Chlorination significantly decreased filamentous bacteria from the genera *Nocardioides* (*p* < 0.01) and *Gordonia* (*p* < 0.05) (Table 5). Apart from *Zoogloea*, all other filamentous bacteria decreased in their relative abundance, although in a not significant manner (Table 5). On the other hand, *Zoogloea* spp. were found to constitute 0.64% of the bacterial community in the disinfected effluent.

*Mycobacterium* spp. disappeared following the application of the disinfectant, and chlorination also reduced the limited number of *Treponema* (Table 6). No strains of the genus *Escherichia* were found. The other potentially pathogenic bacteria identified were detected as a few reads.

Furthermore, the relative abundance of toxic cyanobacteria did not exceed 0.25% of the total bacterial population (Table 7). Notably, *Anabaena* and *Microcystis* accounted for 0.04% and 0.02%, respectively. Although the relative abundance of the toxic cyanobacteria was relatively low, it appeared that its abundance remained unaffected by the amount of hypochlorite applied.

## 4. Discussion

The high BOD, COD and TSS removal efficiencies in the full-scale wastewater treatment plant indicated its efficient operation. Notably, the quality criteria for BOD_5_, COD and TSS, i.e., 25, 125 and 35 mg/L, respectively, set by the Council Directive 91/271/EEC for effluent discharge, were met after the WWTP operation.

Similar to our study, *Aquabacterium* and *Hydrogenophaga* were found to be very sensitive to chlorine in a previous study [21]. Moreover, Pang et al. [8] reported that *Pseudomonas* and *Clostridium* were among the most persistent taxa when secondary effluent was treated with 5 mg Cl_2_/L. *Acidovorax* and *Pseudomonas* are common denitrifies that inhabit activated sludge with a known ability to resist chlorination [33,34,35], indicating that such taxa can be effectively adapted to the oxidative action of hypochlorite. Multiple layers of extracellular polymeric substances (EPS) and biofilm formation are key parameters in the adaptation of such taxa, since the penetration of the disinfectant is prevented [36]. Accordingly, bacterial taxa detected in the current study, such as *Zoogloea*, *Thauera*, *Dechloromonas* and *Candidatus* Accumulibacter, have been reported to harbor gene clusters for EPS synthesis and floc formation in their genomes [37]. Moreover, *Clostridium* and *Bacteroides* spp., which are indicators of human fecal contamination, are considered as chlorination-resistant microorganisms effectively adapted to high doses of disinfectant [23,38]. *Flavobacterium* and *Sphingobacterium* have been also reported to survive disinfection through biofilm formation [39]. In particular, members of the genus *Flavobacterium* are known to persist through chlorination [40]. In addition, the low dissolved oxygen level in the secondary sedimentation tank favored or simply retained the aerotolerant/anaerobic taxa of the intestinal tract, such as the strains of the genera *Bacteroides*, *Arcobacter* and *Clostridium*.

Estimation of the Shannon diversity index showed no significant alterations in the bacterial diversity with the application of hypochlorite. Moreover, Simpson’s index values close to 1 indicated that bacterial communities were highly diverse prior to and after chlorination. On the other hand, Fisher’s diversity index denoted decreased species abundance at the logarithmic scale. Pang et al. [8] reported a decrease in the Chao1 index of the bacterial community in the effluent of some of the examined WWTPs after chlorination, and similar results were also provided by Lin et al. [21]. Indeed, the decrease in Chao1 and Fisher, and not in Shannon and Simpson, indices implies a higher impact of chlorination on the richness and abundance of bacterial taxa rather than on the communities’ evenness, as Pielou’s evenness indexes showed. Moreover, estimation of beta-diversity prior to and after chlorination indicated similar bacterial community structures and minor effects of chlorination on the diversity.

In this study, no statistically significant effect of the addition of hypochlorite on the abundance of members of the phylum *Chloroflexi* with dechlorination capability was observed, indicating the resistance of these microbial constituents to chlorination. (Per)chlorate-reducing bacteria convert perchlorate to chlorate and then to chlorite [31], wherein hypochlorite is formed as the transient intermediate [41,42]. Thus, (per)chlorate-respiring bacteria possess the genetic capability to deal with the toxic effects of hypochlorite. In addition, bacteria from the genera *Dehalobacter*, *Dehalococcoides* and *Dehalogenimonas* are also capable of organochlorine respiration [43,44,45]. As a consequence of chlorination, hypochlorite can react with organic compounds and form organochlorinated derivatives with harmful effects on aquatic organisms and humans. Thus, the relatively high abundance of *Chloroflexi* can be attributed to their dechlorinating ability [46].

Similar to the current study, Tian et al. [47] and Numberger et al. [2] reported the survival of *Candidatus* Accumulibacter after disinfection with ozone and UV radiation, respectively. Apart from the resistance of these uncultured taxa to chlorination, the possibility of reducing (per)chlorate or organochlorinated compounds could not be ruled out. Taking into account that PAOs, performing EBPR, are able to grow in the anoxic zone, and the addition of hypochlorites in the absence of oxygen adjusts redox potential to negative values near zero (anoxic conditions), the ability of such microbiota to reduce perchlorate or organochlorines deserves further investigation. Moreover, nitrifiers of the genera *Nitrosomonas* and *Nitrospira* have been found to proliferate after chlorination [48].

In the present study, the abundance of filamentous bacteria responsible for sludge bulking and foaming can be considered as low in both non-disinfected and disinfected effluent. Filamentous bacteria are the causing agents of bulking and foaming in WWTPs, which can reduce activated sludge sedimentability, thus negatively affecting the water quality of the effluent [49]. Notably, such foams have recently been found to carry antibiotic-resistant genes and human pathogenic bacteria, spreading them in the recipient water bodies [50]. Caravelli et al. [51] reported that chlorination resulted in the inhibition of filamentous bacteria in activated sludge. On the other hand, similarly to our results, *Nocardioides* was found to be among the microbial constituents of a biofilm developed in a chlorinated drinking water distribution system [52]. Besides this, *Zoogloea* spp. are known for their ability to form specific cell aggregates enclosed in gelatinous matrices, a fact that facilitate their resistance to chlorination.

Chlorination is considered as a necessary treatment to significantly reduce the percentage of opportunistic pathogens. Regarding the presence of potentially pathogenic bacteria in the current study, Illumina sequencing revealed that their relative abundance was extremely low, even in the non-disinfected effluent, indicating a low risk from the presence of opportunistic pathogenic bacteria. In particular, no risk from fecal coliform contamination was found in the current case since *Escherichia* strains were not detected. Moreover, *Mycobacterium* spp. disappeared following the application of the disinfectant and a limited number of *Treponema* sequences were detected after chlorination. Similar to our study, *Mycobacterium* was included by Park et al. [23] in the list of the most chlorine-sensitive taxa. The other potentially pathogenic bacteria detected as a few reads are unlikely to present a threat to the ecosystem and human health.

Cyanobacteria, which can secrete toxic substances that may adversely affect the ecology of aquatic organisms and ultimately human health, were also investigated [53]. As in the case of opportunistic pathogenic bacteria, the activated sludge process minimized the risk deriving from the presence of toxic cyanobacteria. *Anabaena* and *Microcystis*, which are considered as the main cyanobacterial taxa-secreting toxins [54,55], were present in negligible abundances. It should also be taken into account that their growth was not favored in the aeration tank, due to the high oxygen supply and increased growth of heterotrophs under aerobic conditions, and to the dark-brown color of the mixed liquor, restricting the photosynthetic activity of cyanobacteria.

Although there is no clear trend as regards the susceptibility of taxa to common disinfection methods, it appears that *Candidatus* Accumulibacter, *Candidatus* Competibacter, *Clostridium*, *Flavobacterium*, *Pseudomonas* and *Simplicispira* are capable of resisting conventional disinfection approaches (Table 8). This indicates that additional treatment methods, such as membrane or advanced oxidation processes systems, may be required in the case of effluent discharge in sensitive recipients.

## 5. Conclusions

Common inhabitants of intestinal tract, i.e., *Bacteroides*, *Arcobacter* and *Clostridium*, were found to survive chlorination and constitute the main microbiota detected in the disinfected effluent. In addition, denitrifiers of the activated sludge systems capable of forming biofilms, such as *Acidovorax*, *Pseudomonas* and *Thauera*, as well as *Chloroflexi* with dechlorination capabilities and bacteria involved in enhanced biological phosphorus removal, such as *Candidatus* Accumulibacter and *Candidatus* Competibacter, were included in the list of chlorination-resistant genera. Chlorination significantly affected the decreased filamentous bacteria of the genera *Nocardioides* and *Gordonia*, whereas *Zoogloea* proliferated in the chlorinated effluent. Perchlorate/chlorate respiration and organochlorine degradation seem to be key processes in the resistance of (per)chlorate- and organochlorine-reducing bacteria, detected in the current study (Table 2), to chlorination. No fecal coliform contamination was observed since *Escherichia* spp. were not detected. *Mycobacterium* spp. were absent, whereas a reduced number of *Treponema* was detected in the chlorinated effluent. Toxin-producing cyanobacteria of the genera *Anabaena* and *Microcystis* were identified in low abundances. This indicates that tertiary treatment methods, which result either in the complete retention or elimination of toxic cyanobacteria, such as membrane or advanced oxidation processes systems, may be more appropriate for improving effluent quality in recipients of eutrophication-sensitive areas.

## Figures and Tables

**Table 1:** Physicochemical characteristics of influent and effluent of the full-scale wastewater treatment plant (WWTP) (Mean ± SE, n = 3).

Parameter	Influent	Effluent	Removal (%)
BOD_5_ (mg/L)	363 ± 5	17.3 ± 0.5	95.2 ± 0.1
COD (mg/L)	626 ± 17	67.1 ± 2.4	89.1 ± 0.4
TSS (mg/L)	311 ± 7	18.9 ± 0.7	93.8 ± 0.2
NO_3_^−^-N (mg/L)	-	9.4 ± 0.5	-

**Table 2:** Relative abundances of (per)chlorate- and organochlorine-reducing bacteria in the effluent of WWTP prior to and after chlorination.

Genus	Prior to Chlorination	After Chlorination	Significance
*Anaerolinea*	0.02 ± 0.01	0.01 ± 0.01	*p* < 0.01
*Bellilinea*	1.42 ± 0.70	0.80 ± 0.34	n.s.
*Caldilinea*	2.19 ± 0.96	1.07 ± 0.52	n.s.
*Dehalobacter*	0.01 ± 0.01	0.01 ± 0.01	n.s.
*Dehalobacterium*	At detection limit	At detection limit	n.s.
*Dehalococcoides*	0.37 ± 0.03	0.24 ± 0.06	n.s.
*Dehalogenimonas*	n.d.	At detection limit	n.s.
*Dechloromonas*	3.17 ± 0.21	1.96 ± 0.36	*p* < 0.05
*Levilinea*	0.02 ± 0.01	0.01 ± 0.01	*p* < 0.05
*Longilinea*	1.25 ± 0.26	1.19 ± 0.35	n.s.

n.d.: not detected; n.s.: not significant.

**Table 3:** Relative abundances of ammonia- and nitrite-oxidizing bacteria in the effluent of WWTP prior to and after chlorination.

Genus	Prior to Chlorination	After Chlorination	Significance
*Candidatus* Nitrotoga	0.03 ± 0.02	0.01 ± 0.01	n.s.
*Nitrobacter*	At detection limit	At detection limit	n.s.
*Nitrosomonas*	0.23 ± 0.20	0.04 ± 0.03	n.s.
*Nitrospira*	0.10 ± 0.03	0.18 ± 0.05	n.s.
*Nitrosovibrio*	0.27 ± 0.03	n.d.	*p* < 0.01

n.d.: not detected; n.s.: not significant.

**Table 4:** Relative abundances of bacterial taxa influencing enhanced biological phosphorus removal and annamox bacteria in the effluent of WWTP prior to and after chlorination.

Genus	Prior to Chlorination	After Chlorination	Significance
**Bacterial Taxa Influencing Enhanced Phosphorus Removal**			
*Candidatus* Accumulibacter	3.31 ± 0.29	2.37 ± 0.63	n.s.
*Candidatus* Competibacter	1.02 ± 0.06	1.02 ± 0.17	n.s.
**Annamox bacteria**			
*Candidatus* Anammoximicrobium	At detection limit	0.01 ± 0.01	n.s.
*Candidatus* Kuenenia	At detection limit	0.01 ± 0.01	n.s.

n.s.: not significant.

**Table 5:** Relative abundances of filamentous bacteria in the effluent of WWTP prior to and after chlorination.

Genus	Prior to Chlorination	After Chlorination	Significance
*Beggiatoa*	0.06 ± 0.03	0.03 ± 0.02	n.s.
*Candidatus* Microthrix	0.47 ± 0.21	0.08 ± 0.04	n.s.
*Gordonia*	0.47 ± 0.05	0.21 ± 0.04	*p* < 0.05
*Haliscomenobacter*	0.77 ± 0.10	0.43 ± 0.15	n.s.
*Nocardioides*	0.96 ± 0.10	0.19 ± 0.04	*p* < 0.01
*Nostocoida* Type II	At detection limit	n.d.	n.s.
*Thiothrix*	0.06 ± 0.01	0.02 ± 0.01	n.s.
*Zoogloea*	0.42 ± 0.02	0.64 ± 0.08	n.s.

n.d.: not detected; n.s.: not significant.

**Table 6:** Relative abundances of potential pathogenic bacteria in the effluent of WWTP prior to and after chlorination.

Genus	Prior Chlorination	After Chlorination	Significance
*Brucella*	0.03 ± 0.02	At detection limit	n.s.
*Campylobacter*	0.02 ± 0.01	0.04 ± 0.02	n.s.
*Enterococcus*	0.01 ± 0.01	0.01 ± 0.01	n.s.
*Klebsiella*	n.d.	At detection limit	n.s.
*Legionella*	0.01 ± 0.01	0.05 ± 0.04	n.s.
*Mycobacterium*	0.15 ± 0.05	n.d.	*p* < 0.05
*Rickettsia*	0.03 ± 0.01	0.40 ± 0.37	n.s.
*Shigella*	At detection limit	n.d.	n.s.
*Spirochaeta*	0.04 ± 0.01	0.07 ± 0.04	n.s.
*Treponema*	0.09 ± 0.02	0.05 ± 0.00	n.s.
*Vibrio*	At detection limit	0.45 ± 0.45	n.s.

n.d.: not detected; n.s.: not significant.

**Table 7:** Relative abundances of cyanobacteria capable of producing toxins in the effluent of WWTP prior to and after chlorination.

Genus	Prior to Chlorination	After Chlorination	Significance
*Anabaena*	n.d.	0.04 ± 0.01	*p* < 0.01
*Arthrospira*	n.d.	At detection limit	n.s.
*Cyanobacterium*	At detection limit	0.01 ± 0.01	n.s.
*Cyanothece*	n.d.	0.01 ± 0.01	*p* < 0.01
*Elstera*	At detection limit	At detection limit	n.s.
*Gloeobacter*	0.08 ± 0.04	0.08 ± 0.03	n.s.
*Leptolyngbya*	0.24 ± 0.16	0.04 ± 0.02	n.s.
*Microcystis*	0.03 ± 0.01	0.02 ± 0.00	n.s.
*Oscillatoria*	At detection limit	At detection limit	n.s.
*Phormidium*	At detection limit	At detection limit	n.s.
*Prochlorococcus*	0.03 ± 0.01	0.03 ± 0.01	n.s.
*Pseudanabaena*	At detection limit	At detection limit	n.s.
*Vampirovibrio*	0.01 ± 0.01	At detection limit	n.s.

n.d.: not detected; n.s.: not significant.

**Table 8:** Effect of disinfection methods on effluent microbiota in municipal WWTPs: susceptible and resistant taxa to disinfection as detected by high-throughput amplicon sequencing.

Disinfection Method	Susceptible Taxa	Resistant Taxa	Reference
Monochloramine	*Arcobacter*, *Nitrospira*, *Sphingobium*	*Chryseobacterium*, *Cloacibacterium*, *Clostridium*, *Mycobacterium*, *Pseudomonas*, *Sphingomonas*, *Streptococcus*, *Undibacterium*	[15]
H_2_O_2_	*Denitratisoma*, *Thauera*	*Anaerolinea*, *Filimonas*	[15]
UV ^1^	*Acetoanaerobium*, *Acidovorax*, *Acinetobacter*, *Aeromonas*, *Anaerosinus*, *Aquabacterium*, *Arcobacter*, *Comamonas*, *Enterococcus*, *Faecalibacterium*, *Paracoccus*, *Proteocatella*, *Streptococcus*, *Subdoligranulum*, *Trichococcus*, *Uruburuella*, *Veillonella*	*Ca.* Accumulibacter, *Ca*. Competibacter, *Ca*. Nitrotoga, *Chryseobacterium*, *Cupriavidus*, *Dechloromonas*, *Geothrix*, *Nitrosomonas*, *Nitrospira*, *Rhodoferax*, *Simplicispira*, *Thauera*, *Zoogloea*	[2]
UV-C/H_2_O_2_/IDS-Cu ^2^	N.R.	*Acinetobacter*, *Pantoea*, *Pseudomonas*	[16]
Chlorination ^4^	*Acinetobacter*, *Arcobacter*, *Azonexus*, *Azospira*, *Bifidobacterium*, *Chitinimonas*, *Comamonas*, *Dechloromonas*, *Enterococcus*, *Lactobacillales*, *Laribacter*, *Neisseria*, *Nitrosomonas*, *Propionivibrio*, *Rheinheimera*, *Salmonella*, *Shewanella*, *Thauera*, *Tolumonas*, *Vitreoscilla*, *Zoogloea*	*Aquabacterium*, *Chryseobacterium*, *Clostridium*, *Flavobacterium*, *Gemmata*, *Hydrogenophaga*, *Legionella*, *Mycobacterium*, *Pseudomonas*, *Rubrivivax*, *Turicibacter*, *Veillonella*	[3,8]
Chlorination	*Aeromicrobium*, *Aquabacterium*, *Dechloromonas*, *Eubacterium*, *Hydrogenophaga*^3^	*Acidovorax*, *Arcobacter*, *Azospirillum*, *Bacteroides*, *Bellilinea*, *Brevundimonas*, *Caldilinea*, *Ca*. Accumulibacter, *Ca*. Competibacter, *Clostridium*, *Curvibacter*, *Dietzia*, *Flavobacterium*, *Hahella*, *Lapillicoccus*, *Longilinea*, *Malikia*, *Polaromonas*, *Pseudomonas*, *Ruminococcus*, *Runella*, *Simplicispira*, *Sphingobacterium*, *Streptomyces*, *Thauera*, *Thiococcus*^3^	This study

^1^, municipal wastewater plus minor percentage of industrial wastewater; ^2^, IDS-Cu, iminodisuccinic acid complex; ^3^, only major taxa reported; N.R., not reported; ^4^, *Aeromonas* was reported as susceptible and resistant by Pang et al. [8] and Greay et al. [3], respectively, whereas the opposite was reported for *Corynebacterium* and *Streptococcus* by the same authors.

## Supplementary Materials

The following is available online at https://www.mdpi.com/2075-1729/11/1/68/s1, Table S1: Minor taxa identified in the effluent of the full-scale municipal WWTP prior to and after chlorination.
