# Supplementary material for: Effect of Chlorination on Microbiological Quality of Effluent of a Full-Scale Wastewater Treatment Plant
Source: Life (Basel). 2021 Jan 19;11(1):68. doi: 10.3390/life11010068 (PMC7832327; doi:10.3390/life11010068)
Supplement: Supplementary file 1 [file life-11-00068-s001.pdf]

Supplementary Table S1. Minor taxa identified in the effluent of the full-scale municipal WWTP prior and after chlorination.

Genus	Prior chlorination	After chlorination	*p*-value[1,2]	Genus	Prior chlorination	After chlorination	*p*-value[1,2]
Nocardioides	0.96 ± 0.10	0.18 ± 0.04	*0.01*	*Caulobacter*	0.34 ± 0.20	0.12 ± 0.10	n.s.
Fusobacterium	0.91 ± 0.83	1.06 ± 1.03	n.s.	*Thiobacillus*	0.31 ± 0.07	0.19 ± 0.05	n.s.
Isosphaera	0.85 ± 0.54	1.18 ± 1.06	n.s.	*Sphingomonas*	0.30 ± 0.05	0.10 ± 0.07	n.s.
Afipia	0.85 ± 0.32	0.12 ± 0.01	n.s.	*Pseudoxanthomonas*	0.30 ± 0.04	0.03 ± 0.01	*0.01*
Sporichthya	0.85 ± 0.16	0.44 ± 0.04	n.s.	*Fibrobacter*	0.28 ± 0.10	0.39 ± 0.11	n.s.
Mariniphaga	0.85 ± 0.35	0.96 ± 0.70	n.s.	*Ottowia*	0.28 ± 0.04	0.15 ± 0.04	n.s.
Ilumatobacter	0.85 ± 0.52	0.23 ± 0.13	n.s.	*Fluviicola*	0.27 ± 0.01	0.32 ± 0.10	n.s.
Planctomyces	0.79 ± 0.63	0.64 ± 0.52	n.s.	*Nitrosovibrio*	0.26 ± 0.03	0.24 ± 0.01	n.s.
Haliscomenobacter	0.77 ± 0.10	0.42 ± 0.15	n.s.	*Leucobacter*	0.26 ± 0.01	0.23 ± 0.06	n.s.
Lewinella	0.73 ± 0.39	0.66 ± 0.57	n.s.	*Xanthomonas*	0.24 ± 0.05	0.12 ± 0.04	n.s.
Rhodobacter	0.69 ± 0.16	0.23 ± 0.05	n.s.	*Variovorax*	0.24 ± 0.04	0.04 ± 0.04	*0.05*
Bacillus	0.64 ± 0.09	0.50 ± 0.23	n.s.	*Acinetobacter*	0.24 ± 0.15	0.75 ± 0.52	n.s.
Conexibacter	0.62 ± 0.37	0.25 ± 0.21	n.s.	*Oligotropha*	0.24 ± 0.15	0.08 ± 0.07	n.s.
Rhodomicrobium	0.62 ± 0.07	0.35 ± 0.12	n.s.	*Leptolyngbya*	0.24 ± 0.16	0.04 ± 0.02	n.s.
Prosthecobacter	0.60 ± 0.16	0.30 ± 0.17	n.s.	*Iamia*	0.23 ± 0.16	0.11 ± 0.10	n.s.
Rhodococcus	0.58 ± 0.11	0.07 ± 0.01	*0.01*	*Nitrosomonas*	0.22 ± 0.20	0.04 ± 0.03	n.s.
Pedobacter	0.57 ± 0.21	0.33 ± 0.27	n.s.	*Ideonella*	0.22 ± 0.09	0.02 ± 0.01	n.s.
Pirellula	0.56 ± 0.39	0.48 ± 0.39	n.s.	*Tetrasphaera*	0.21 ± 0.03	0.08 ± 0.04	n.s.
Saccharibacter	0.53 ± 0.06	0.26 ± 0.09	n.s.	*Novosphingobium*	0.21 ± 0.07	0.07 ± 0.05	n.s.
Bdellovibrio	0.53 ± 0.23	0.93 ± 0.75	n.s.	*Ignavibacterium*	0.20 ± 0.02	0.25 ± 0.12	n.s.
Rhodocyclus	0.52 ± 0.05	0.42 ± 0.05	n.s.	*Hyphomicrobium*	0.20 ± 0.05	0.09 ± 0.05	n.s.
Bosea	0.50 ± 0.23	0.05 ± 0.02	n.s.	*Gemmobacter*	0.20 ± 0.02	0.08 ± 0.03	*0.05*
Phenylobacterium	0.48 ± 0.20	0.06 ± 0.01	n.s.	*Moorella*	0.19 ± 0.13	0.03 ± 0.02	n.s.
Trichococcus	0.48 ± 0.11	0.51 ± 0.14	n.s.	*Rhizobium*	0.19 ± 0.02	0.13 ± 0.07	n.s.
Ca. Microthrix	0.47 ± 0.21	0.09 ± 0.04	n.s.	*Faecalibacterium*	0.19 ± 0.07	0.19 ± 0.10	n.s.
Gordonia	0.46 ± 0.05	0.21 ± 0.04	*0.05*	*Nitratireductor*	0.18 ± 0.03	0.03 ± 0.02	*0.05*
Opitutus	0.46 ± 0.19	0.46 ± 0.31	n.s.	*Methylophilus*	0.18 ± 0.02	0.26 ± 0.11	n.s.
Cytophaga	0.44 ± 0.03	0.65 ± 0.21	n.s.	*Ca.* Saccharimonas	0.17 ± 0.03	0.17 ± 0.06	n.s.
Methylocella	0.43 ± 0.09	0.23 ± 0.12	n.s.	*Pedosphaera*	0.17 ± 0.07	0.40 ± 0.26	n.s.
Desulfomicrobium	0.43 ± 0.07	0.53 ± 0.10	n.s.	*Cloacibacillus*	0.17 ± 0.02	0.32 ± 0.12	n.s.
Zoogloea	0.42 ± 0.01	0.64 ± 0.08	n.s.	*Polyangium*	0.17 ± 0.09	0.15 ± 0.13	n.s.
Methyloversatilis	0.42 ± 0.01	0.22 ± 0.06	*0.05*	*Chelatococcus*	0.17 ± 0.09	0.07 ± 0.06	n.s.
Paludibacter	0.41 ± 0.19	0.88 ± 0.37	n.s.	*Lutimaribacter*	0.16 ± 0.01	0.00 ± 0.00	*0.01*
Lactivibrio	0.40 ± 0.18	0.26 ± 0.09	n.s.	*Geobacter*	0.16 ± 0.04	0.14 ± 0.02	n.s.
Chthoniobacter	0.40 ± 0.10	0.66 ± 0.03	n.s.	*Gemmata*	0.16 ± 0.05	0.14 ± 0.03	n.s.
Perlucidibaca	0.39 ± 0.15	0.18 ± 0.05	n.s.	*Gemmatimonas*	0.16 ± 0.14	0.07 ± 0.07	n.s.
Dehalococcoides	0.37 ± 0.03	0.24 ± 0.06	n.s.	*Derxia*	0.16 ± 0.05	0.09 ± 0.07	n.s.
Victivallis	0.37 ± 0.17	0.47 ± 0.19	n.s.	*Comamonas*	0.15 ± 0.04	0.18 ± 0.07	n.s.
Paracoccus	0.36 ± 0.06	0.11 ± 0.06	*0.05*	*Rhodopirellula*	0.15 ± 0.05	0.08 ± 0.04	n.s.
Prosthecomicrobium	0.36 ± 0.10	0.17 ± 0.10	n.s.	*Mycobacterium*	0.15 ± 0.05	0.16 ± 0.13	n.s.
Pelosinus	0.35 ± 0.14	0.57 ± 0.18	n.s.	*Roseomonas*	0.15 ± 0.06	0.06 ± 0.02	n.s.
Verrucomicrobium	0.34 ± 0.05	0.42 ± 0.06	n.s.	*Devosia*	0.14 ± 0.10	0.03 ± 0.01	n.s.

Genus	Prior chlorination	After chlorination	*p*-value[1,2]	Genus	Prior chlorination	After chlorination	*p*-value[1,2]
Bacteriovorax	0.14 ± 0.05	0.20 ± 0.10	n.s.	*Saccharofermentans*	0.07 ± 0.03	0.05 ± 0.04	n.s.
Thioflavicoccus	0.14 ± 0.00	0.10 ± 0.01	n.s.	*Xanthobacter*	0.07 ± 0.02	0.02 ± 0.01	*0.05*
Nannocystis	0.13 ± 0.12	0.19 ± 0.18	n.s.	*Sphaerobacter*	0.07 ± 0.03	0.04 ± 0.03	n.s.
Sterolibacterium	0.13 ± 0.06	0.04 ± 0.03	n.s.	*Desulfuromusa*	0.07 ± 0.03	0.05 ± 0.04	n.s.
Crocinitomix	0.13 ± 0.08	0.06 ± 0.05	n.s.	*Coraliomargarita*	0.07 ± 0.06	0.29 ± 0.19	n.s.
Agitococcus	0.13 ± 0.07	0.05 ± 0.02	n.s.	*Azospira*	0.06 ± 0.01	0.01 ± 0.01	*0.05*
Geothermobacter	0.12 ± 0.03	0.82 ± 0.80	n.s.	*Thiothrix*	0.06 ± 0.01	0.02 ± 0.01	n.s.
Natronoanaerobium	0.12 ± 0.07	0.07 ± 0.06	n.s.	*Ferrovum*	0.06 ± 0.02	0.02 ± 0.00	n.s.
Chthonomonas	0.12 ± 0.06	0.10 ± 0.03	n.s.	*Cupriavidus*	0.06 ± 0.05	0.22 ± 0.13	n.s.
Desulfobulbus	0.12 ± 0.02	0.24 ± 0.05	n.s.	*Beggiatoa*	0.06 ± 0.03	0.03 ± 0.02	n.s.
Chloroflexus	0.11 ± 0.05	0.06 ± 0.01	n.s.	*Acidithiobacillus*	0.06 ± 0.02	0.05 ± 0.02	n.s.
Acidomonas	0.11 ± 0.06	0.07 ± 0.06	n.s.	*Thermomonas*	0.06 ± 0.01	0.01 ± 0.00	*0.01*
Lutibacter	0.11 ± 0.05	0.02 ± 0.01	n.s.	*Oceanicola*	0.06 ± 0.02	0.03 ± 0.01	n.s.
Rhodoferax	0.11 ± 0.02	0.18 ± 0.04	n.s.	*Rhodoplanes*	0.06 ± 0.01	0.03 ± 0.01	n.s.
Aminobacterium	0.11 ± 0.05	0.00 ± 0.00	n.s.	*Aquaspirillum*	0.06 ± 0.05	0.42 ± 0.24	n.s.
Mesorhizobium	0.11 ± 0.05	0.04 ± 0.02	n.s.	*Phycicoccus*	0.06 ± 0.00	0.00 ± 0.00	*0.01*
Rhodovastum	0.10 ± 0.06	0.04 ± 0.04	n.s.	*Bifidobacterium*	0.06 ± 0.01	0.14 ± 0.06	n.s.
Sorangium	0.10 ± 0.07	0.11 ± 0.10	n.s.	*Janibacter*	0.05 ± 0.01	0.01 ± 0.01	*0.05*
Neochlamydia	0.10 ± 0.04	0.17 ± 0.06	n.s.	*Aeromonas*	0.05 ± 0.04	0.04 ± 0.03	n.s.
Steroidobacter	0.10 ± 0.06	0.07 ± 0.03	n.s.	*Desulfovibrio*	0.05 ± 0.02	0.10 ± 0.03	n.s.
Anaerovorax	0.10 ± 0.02	0.22 ± 0.08	n.s.	*Ca.* Koribacter	0.05 ± 0.05	0.04 ± 0.04	n.s.
Stella	0.10 ± 0.04	0.08 ± 0.05	n.s.	*Pleomorphomonas*	0.05 ± 0.04	0.02 ± 0.02	n.s.
Nitrospira	0.10 ± 0.03	0.18 ± 0.05	n.s.	*Geoalkalibacter*	0.05 ± 0.05	0.02 ± 0.02	n.s.
Sulfurospirillum	0.10 ± 0.05	0.12 ± 0.08	n.s.	*Turneriella*	0.05 ± 0.05	0.14 ± 0.13	n.s.
Treponema	0.09 ± 0.02	0.05 ± 0.01	n.s.	*Leadbetterella*	0.05 ± 0.03	0.01 ± 0.00	n.s.
Polynucleobacter	0.09 ± 0.02	0.07 ± 0.03	n.s.	*Selenomonas*	0.05 ± 0.02	0.11 ± 0.04	n.s.
Ca. Odyssella	0.09 ± 0.04	0.09 ± 0.06	n.s.	*Desulforegula*	0.05 ± 0.01	0.05 ± 0.01	n.s.
Synergistes	0.09 ± 0.02	0.07 ± 0.02	n.s.	*Prolixibacter*	0.05 ± 0.01	0.04 ± 0.03	n.s.
Microterricola	0.09 ± 0.03	0.04 ± 0.03	n.s.	*Amphiplicatus*	0.05 ± 0.00	0.00 ± 0.00	*0.01*
Defluviicoccus	0.09 ± 0.05	0.04 ± 0.03	n.s.	*Aciditerrimonas*	0.05 ± 0.04	0.03 ± 0.02	n.s.
Fulvivirga	0.09 ± 0.04	0.06 ± 0.03	n.s.	*Pseudacidovorax*	0.04 ± 0.01	0.02 ± 0.01	n.s.
Achromobacter	0.09 ± 0.03	0.06 ± 0.03	n.s.	*Spirochaeta*	0.04 ± 0.01	0.07 ± 0.04	n.s.
Streptococcus	0.09 ± 0.03	0.11 ± 0.06	n.s.	*Intestinibacter*	0.04 ± 0.00	0.01 ± 0.00	*0.01*
Microbacterium	0.09 ± 0.01	0.07 ± 0.01	n.s.	*Oscillospira*	0.04 ± 0.01	0.03 ± 0.02	n.s.
Methylosinus	0.08 ± 0.04	0.03 ± 0.02	n.s.	*Sandaracinus*	0.04 ± 0.02	0.08 ± 0.08	n.s.
Beijerinckia	0.08 ± 0.01	0.03 ± 0.01	*0.01*	*Turicibacter*	0.04 ± 0.01	0.03 ± 0.01	n.s.
Flexibacter	0.08 ± 0.03	0.11 ± 0.07	n.s.	*Acetobacterium*	0.04 ± 0.01	0.06 ± 0.01	n.s.
Chitinimonas	0.08 ± 0.01	0.00 ± 0.00	*0.01*	*Blautia*	0.04 ± 0.02	0.04 ± 0.02	n.s.
Poseidonocella	0.07 ± 0.01	0.00 ± 0.00	*0.01*	*Roseburia*	0.04 ± 0.02	0.02 ± 0.01	n.s.
Gloeobacter	0.07 ± 0.04	0.08 ± 0.03	n.s.	*Stenotrophomonas*	0.04 ± 0.01	0.05 ± 0.01	n.s.
Kofleria	0.07 ± 0.05	0.05 ± 0.04	n.s.	*Dysgonomonas*	0.04 ± 0.02	0.07 ± 0.03	n.s.
Leifsonia	0.07 ± 0.02	0.02 ± 0.01	n.s.	*Providencia*	0.04 ± 0.01	0.01 ± 0.00	n.s.
Dongia	0.07 ± 0.03	0.04 ± 0.03	n.s.	*Leptospira*	0.04 ± 0.01	0.05 ± 0.01	n.s.
Spirulina	0.07 ± 0.05	0.03 ± 0.02	n.s.	*Alistipes*	0.04 ± 0.02	0.08 ± 0.05	n.s.
Chlorobium	0.07 ± 0.03	0.07 ± 0.01	n.s.	*Thermaerobacter*	0.04 ± 0.02	0.02 ± 0.01	n.s.

Genus	Prior chlorination	After chlorination	*p*-value[1,2]	Genus	Prior chlorination	After chlorination	*p*-value[1,2]
Compostimonas	0.04 ± 0.01	0.23 ± 0.20	n.s.	*Oxalobacter*	0.02 ± 0.01	0.00 ± 0.00	n.s.
Algisphaera	0.04 ± 0.00	0.01 ± 0.00	*0.01*	*Sphingopyxis*	0.02 ± 0.01	0.00 ± 0.00	n.s.
Blastopirellula	0.04 ± 0.01	0.07 ± 0.01	n.s.	*Ca.* Nitrotoga	0.02 ± 0.02	0.01 ± 0.01	n.s.
Blastochloris	0.04 ± 0.01	0.01 ± 0.01	n.s.	*Tolumonas*	0.02 ± 0.02	0.02 ± 0.01	n.s.
Limnobacter	0.04 ± 0.01	0.03 ± 0.01	n.s.	*Chondromyces*	0.02 ± 0.02	0.01 ± 0.01	n.s.
Microvirga	0.04 ± 0.02	0.08 ± 0.08	n.s.	*Leptothrix*	0.02 ± 0.01	0.04 ± 0.02	n.s.
Acetivibrio	0.04 ± 0.01	0.04 ± 0.01	n.s.	*Geothrix*	0.02 ± 0.01	0.01 ± 0.01	n.s.
Microcystis	0.03 ± 0.01	0.02 ± 0.00	n.s.	*Ca.* Cloacimonas	0.02 ± 0.01	0.00 ± 0.00	n.s.
Methylocystis	0.03 ± 0.01	0.01 ± 0.01	n.s.	*Ectothiorhodospira*	0.02 ± 0.00	0.00 ± 0.00	*0.01*
Roseobacter	0.03 ± 0.01	0.02 ± 0.01	n.s.	*Sphingorhabdus*	0.02 ± 0.00	0.00 ± 0.00	*0.01*
Rickettsia	0.03 ± 0.01	0.40 ± 0.37	n.s.	*Quatrionicoccus*	0.02 ± 0.01	0.19 ± 0.10	n.s.
Dyadobacter	0.03 ± 0.03	0.00 ± 0.00	n.s.	*Anoxybacillus*	0.02 ± 0.01	0.09 ± 0.03	n.s.
Myxobacter	0.03 ± 0.03	0.00 ± 0.00	n.s.	*Rhodopseudomonas*	0.02 ± 0.00	0.01 ± 0.00	*0.05*
Nordella	0.03 ± 0.01	0.00 ± 0.00	*0.05*	*Campylobacter*	0.02 ± 0.01	0.04 ± 0.02	n.s.
Ochrobactrum	0.03 ± 0.01	0.01 ± 0.01	n.s.	*Cryobacterium*	0.02 ± 0.01	0.00 ± 0.00	*0.05*
Kaistia	0.03 ± 0.01	0.01 ± 0.00	n.s.	*Marinobacterium*	0.02 ± 0.00	0.00 ± 0.00	n.a.
Wolbachia	0.03 ± 0.03	0.00 ± 0.00	n.s.	*Sanguibacter*	0.02 ± 0.00	0.00 ± 0.00	n.a.
Oligosphaera	0.03 ± 0.02	0.03 ± 0.01	n.s.	*Alcaligenes*	0.02 ± 0.01	0.01 ± 0.01	n.s.
Rheinheimera	0.03 ± 0.01	0.20 ± 0.08	n.s.	*Methylocaldum*	0.02 ± 0.00	0.02 ± 0.01	n.s.
Ca. Magnetobacterium	0.03 ± 0.01	0.03 ± 0.00	n.s.	*Citreicella*	0.02 ± 0.01	0.00 ± 0.00	*0.05*
Terrimonas	0.03 ± 0.02	0.02 ± 0.01	n.s.	*Microbulbifer*	0.02 ± 0.00	0.03 ± 0.00	*0.05*
Prochlorococcus	0.03 ± 0.01	0.03 ± 0.00	n.s.	*Collinsella*	0.02 ± 0.01	0.01 ± 0.01	n.s.
Anaeromyxobacter	0.03 ± 0.02	0.02 ± 0.02	n.s.	*Halobacillus*	0.02 ± 0.00	0.00 ± 0.00	n.a.
Sporanaerobacter	0.03 ± 0.01	0.05 ± 0.04	n.s.	*Methylibium*	0.02 ± 0.01	0.01 ± 0.01	n.s.
Anaerophaga	0.03 ± 0.01	0.03 ± 0.01	n.s.	*Sphingosinicella*	0.02 ± 0.01	0.01 ± 0.01	n.s.
Rikenella	0.03 ± 0.01	0.00 ± 0.00	n.s.	*Elusimicrobium*	0.02 ± 0.02	0.03 ± 0.03	n.s.
Actinomyces	0.03 ± 0.00	0.01 ± 0.01	n.s.	*Phaselicystis*	0.02 ± 0.02	0.01 ± 0.01	n.s.
Leptotrichia	0.03 ± 0.01	0.02 ± 0.01	n.s.	*Sediminibacterium*	0.02 ± 0.02	0.01 ± 0.01	n.s.
Pseudorhodoferax	0.03 ± 0.00	0.00 ± 0.00	*0.01*	*Ramlibacter*	0.02 ± 0.00	0.04 ± 0.03	n.s.
Propionigenium	0.03 ± 0.02	0.03 ± 0.02	n.s.	*Holdemanella*	0.02 ± 0.00	0.03 ± 0.02	n.s.
Cetobacterium	0.03 ± 0.02	0.02 ± 0.01	n.s.	*Dorea*	0.02 ± 0.01	0.02 ± 0.01	n.s.
Aquiflexum	0.03 ± 0.01	0.04 ± 0.01	n.s.	*Rhodobium*	0.02 ± 0.00	0.01 ± 0.00	n.s.
Brucella	0.03 ± 0.02	0.00 ± 0.00	n.s.	*Alkaliflexus*	0.02 ± 0.00	0.03 ± 0.01	n.s.
Thiorhodospira	0.03 ± 0.02	0.03 ± 0.02	n.s.	*Deferribacter*	0.02 ± 0.01	0.01 ± 0.01	n.s.
Ca. Amoebophilus	0.02 ± 0.01	0.02 ± 0.01	n.s.	*Levilinea*	0.02 ± 0.00	0.01 ± 0.00	n.s.
Kordia	0.02 ± 0.01	0.00 ± 0.00	n.s.	*Marinobacter*	0.02 ± 0.00	0.00 ± 0.00	*0.05*
Anaerolinea	0.02 ± 0.00	0.01 ± 0.00	*0.05*	*Brooklawnia*	0.02 ± 0.00	0.01 ± 0.00	n.s.
Lentisphaera	0.02 ± 0.01	0.02 ± 0.01	n.s.	*Frankia*	0.02 ± 0.00	0.01 ± 0.00	n.s.
Lactobacillus	0.02 ± 0.01	0.02 ± 0.01	n.s.	*Trabulsiella*	0.02 ± 0.00	0.00 ± 0.00	*0.01*
Formosa	0.02 ± 0.00	0.00 ± 0.00	*0.01*	*Paraperlucidibaca*	0.02 ± 0.01	0.02 ± 0.01	n.s.
Bradyrhizobium	0.02 ± 0.00	0.00 ± 0.00	*0.01*	*Sulfuritalea*	0.02 ± 0.01	0.01 ± 0.01	n.s.
Chitinophaga	0.02 ± 0.00	0.01 ± 0.00	*0.05*	*Denitratisoma*	0.02 ± 0.01	0.00 ± 0.00	n.s.
Arthrobacter	0.02 ± 0.01	0.03 ± 0.01	n.s.	*Acidobacterium*	0.02 ± 0.01	0.03 ± 0.01	n.s.
Magnetospirillum	0.02 ± 0.01	0.00 ± 0.00	n.s.	*Sulfurovum*	0.02 ± 0.01	0.01 ± 0.01	n.s.
Litorilinea	0.02 ± 0.01	0.01 ± 0.01	n.s.	*Sulfurimonas*	0.02 ± 0.01	0.01 ± 0.01	n.s.

Genus	Prior chlorination	After chlorination	*p*-value[1,2]	Genus	Prior chlorination	After chlorination	*p*-value[1,2]
Burkholderia	0.02 ± 0.01	0.01 ± 0.01	n.s.	*Dokdonella*	0.01 ± 0.00	0.00 ± 0.00	n.a.
Proteocatella	0.02 ± 0.01	0.03 ± 0.02	n.s.	*Porphyrobacter*	0.01 ± 0.00	0.00 ± 0.00	n.s.
Ralstonia	0.02 ± 0.01	0.02 ± 0.00	n.s.	*Brachymonas*	0.01 ± 0.00	0.00 ± 0.00	n.a.
Haliangium	0.02 ± 0.02	0.01 ± 0.01	n.s.	*Pseudoclavibacter*	0.01 ± 0.00	0.00 ± 0.00	n.a.
Alicyclobacillus	0.02 ± 0.02	0.00 ± 0.00	n.s.	*Vampirovibrio*	0.01 ± 0.00	0.00 ± 0.00	n.s.
Emticicia	0.01 ± 0.01	0.01 ± 0.01	n.s.	*Tessaracoccus*	0.01 ± 0.00	0.00 ± 0.00	n.a.
Erysipelothrix	0.01 ± 0.00	0.02 ± 0.00	n.s.	*Pseudoruegeria*	0.01 ± 0.00	0.00 ± 0.00	n.a.
Truepera	0.01 ± 0.00	0.00 ± 0.00	*0.05*	*Ruminiclostridium*	0.01 ± 0.01	0.01 ± 0.01	n.s.
Phascolarctobacterium	0.01 ± 0.01	0.01 ± 0.01	n.s.	*Thermoanaerobacter*	0.01 ± 0.00	0.00 ± 0.00	n.a.
Azonexus	0.01 ± 0.01	0.02 ± 0.01	n.s.	*Akkermansia*	0.01 ± 0.01	0.02 ± 0.02	n.s.
Pelobacter	0.01 ± 0.00	0.00 ± 0.00	n.s.	*Phaeospirillum*	0.01 ± 0.01	0.01 ± 0.01	n.s.
Rhodovibrio	0.01 ± 0.00	0.03 ± 0.00	*0.05*	*Paracraurococcus*	0.01 ± 0.01	0.00 ± 0.00	n.s.
Roseovarius	0.01 ± 0.00	0.00 ± 0.00	*0.05*	*Phyllobacterium*	0.01 ± 0.01	0.01 ± 0.01	n.s.
Thermodesulfobium	0.01 ± 0.01	0.02 ± 0.01	n.s.	*Legionella*	0.01 ± 0.01	0.05 ± 0.04	n.s.
Catabacter	0.01 ± 0.00	0.00 ± 0.00	n.s.	*Kopriimonas*	0.01 ± 0.01	0.02 ± 0.02	n.s.
Telmatospirillum	0.01 ± 0.00	0.00 ± 0.00	n.s.	*Massilia*	0.01 ± 0.01	0.00 ± 0.00	n.s.
Lachnoclostridium	0.01 ± 0.01	0.01 ± 0.01	n.s.	*Butyrivibrio*	0.01 ± 0.00	0.01 ± 0.01	n.s.
Propionicimonas	0.01 ± 0.00	0.01 ± 0.00	n.s.	*Dehalobacter*	0.01 ± 0.00	0.02 ± 0.01	n.s.
Desulfocaldus	0.01 ± 0.00	0.00 ± 0.00	n.s.	*Brevinema*	0.01 ± 0.00	0.01 ± 0.00	n.s.
Ferribacterium	0.01 ± 0.01	0.00 ± 0.00	n.s.	*Pelotomaculum*	0.01 ± 0.00	0.01 ± 0.00	n.s.
Hyphomonas	0.01 ± 0.01	0.58 ± 0.58	n.s.	*Acetobacter*	0.01 ± 0.00	0.01 ± 0.01	n.s.
Lysobacter	0.01 ± 0.00	0.00 ± 0.00	*0.05*	*Syntrophomonas*	0.01 ± 0.00	0.02 ± 0.01	n.s.
Alkalibacterium	0.01 ± 0.01	0.00 ± 0.00	n.s.	*Adhaeribacter*	0.01 ± 0.00	0.00 ± 0.00	n.s.
Alpinimonas	0.01 ± 0.01	0.00 ± 0.00	n.s.	*Chryseobacterium*	0.01 ± 0.00	0.01 ± 0.01	n.s.
Rhodoligotrophos	0.01 ± 0.01	0.01 ± 0.01	n.s.	*Xanthobacillum*	0.01 ± 0.00	0.01 ± 0.00	n.s.
Insolitispirillum	0.01 ± 0.01	0.00 ± 0.00	n.s.	*Enterococcus*	0.01 ± 0.00	0.01 ± 0.01	n.s.
Pseudaminobacter	0.01 ± 0.01	0.00 0.00	n.s.	*Desulfobacter*	0.01 ± 0.00	0.03 ± 0.02	n.s.
Giesbergeria	0.01 ± 0.01	0.19 ± 0.09	n.s.	*Tyzzerella*	0.01 ± 0.00	0.00 ± 0.00	n.s.
Herbaspirillum	0.01 ± 0.01	0.01 ± 0.00	n.s.	*Gelria*	0.01 ± 0.00	0.00 ± 0.00	n.s.
Muricauda	0.01 ± 0.01	0.01 ± 0.01	n.s.	*Chelativorans*	0.01 ± 0.00	0.00 ± 0.00	n.s.
Fusibacter	0.01 ± 0.01	0.02 ± 0.01	n.s.	*Methylobacillus*	0.01 ± 0.00	0.00 ± 0.00	n.s.
Pelagibacterium	0.01 ± 0.01	0.00 ± 0.00	n.s.	*Thermus*	0.01 ± 0.00	0.02 ± 0.00	*0.05*
Sedimentibacter	0.01 ± 0.01	0.02 ± 0.01	n.s.	*Halomonas*	0.01 ± 0.00	0.00 ± 0.00	n.s.
Desulfuromonas	0.01 ± 0.00	0.01 ± 0.00	n.a.	*Methylotenera*	0.01 ± 0.00	0.60 ± 0.26	n.s.
Marinifilum	0.01 ± 0.00	0.01 ± 0.00	n.a.	*Nevskia*	0.01 ± 0.00	0.01 ± 0.01	n.s.
Georgfuchsia	0.01 ± 0.00	0.00 ± 0.00	n.a.	*Allochromatium*	0.01 ± 0.00	0.00 ± 0.00	n.s.
Limnohabitans	0.01 ± 0.00	0.01 ± 0.01	n.s.	*Aminomonas*	0.01 ± 0.00	0.00 ± 0.00	n.s.
Cystobacter	0.01 ± 0.00	0.01 ± 0.00	n.s.	*Peptoclostridium*	0.01 ± 0.00	0.00 ± 0.00	n.s.
Bauldia	0.01 ± 0.01	0.00 ± 0.00	n.s.	*Prevotella*	0.01 ± 0.00	0.04 ± 0.04	n.s.
Coprococcus	0.01 ± 0.01	0.01 ± 0.01	n.s.	*Dialister*	0.01 ± 0.00	0.00 ± 0.00	n.s.
Fusicatenibacter	0.01 ± 0.01	0.01 ± 0.00	n.s.	*Maricaulis*	0.01 ± 0.00	0.00 ± 0.00	n.s.
Pimelobacter	0.01 ± 0.01	0.00 ± 0.00	n.s.	*Niabella*	0.01 ± 0.00	0.00 ± 0.00	n.s.
Propionibacterium	0.01 ± 0.00	0.00 ± 0.00	n.s.	*Rhodovulum*	0.01 ± 0.00	0.00 ± 0.00	n.s.
Parabacteroides	0.01 ± 0.01	0.04 ± 0.02	n.s.	*Senegalimassilia*	0.01 ± 0.00	0.00 ± 0.00	n.s.
Symbiobacterium	0.01 ± 0.01	0.02 ± 0.01	n.s.	*Sinorhizobium*	0.01 ± 0.00	0.00 ± 0.00	n.s.

Genus	Prior chlorination	After chlorination	*p*-value[1,2]	Genus	Prior chlorination	After chlorination	*p*-value[1,2]
Eggerthella	0.01 ± 0.01	0.01 ± 0.01	n.s.	*Paenibacillus*	0.00 ± 0.00	0.01 ± 0.01	n.s.
Ilyobacter	0.01 ± 0.01	0.01 ± 0.01	n.s.	*Cronobacter*	0.00 ± 0.00	0.01 ± 0.01	n.s.
Arenimonas	0.01 ± 0.01	0.00 ± 0.00	n.s.	*Delftia*	0.00 ± 0.00	0.01 ± 0.00	*0.05*
*Ca.*Phytoplasma	0.01 ± 0.01	0.00 ± 0.00	n.s.	*Desulfobacterium*	0.00 ± 0.00	0.01 ± 0.00	n.s.
Solirubrobacter	0.01 ± 0.00	0.00 ± 0.00	n.s.	*Amaricoccus*	0.00 ± 0.00	0.01 ± 0.00	n.s.
Methylobacterium	0.01 ± 0.00	0.00 ± 0.00	n.s.	*Spirosoma*	0.00 ± 0.00	0.01 ± 0.01	n.s.
Microlunatus	0.01 ± 0.00	0.00 ± 0.00	n.s.	*Alishewanella*	0.00 ± 0.00	0.01 ± 0.00	n.s.
Undibacterium	0.01 ± 0.01	0.02 ± 0.01	n.s.	*Anabaena*	0.00 ± 0.00	0.04 ± 0.00	n.a.
Dermacoccus	0.01 ± 0.00	0.00 ± 0.00	n.s.	*Anditalea*	0.00 ± 0.00	0.01 ± 0.01	n.s.
Laribacter	0.01 ± 0.01	0.01 ± 0.01	n.s.	*Aquimonas*	0.00 ± 0.00	0.01 ± 0.00	n.a.
Aquamicrobium	0.01 ± 0.01	0.00 ± 0.00	n.s.	*Armatimonas*	0.00 ± 0.00	0.02 ± 0.01	*0.05*
Oligoflexus	0.01 ± 0.01	0.01 ± 0.01	n.s.	*Ca.* Hodgkinia	0.00 ± 0.00	0.02 ± 0.02	n.s.
Desulfobacula	0.00 ± 0.00	0.01 ± 0.01	n.s.	*Cyanothece*	0.00 ± 0.00	0.01 ± 0.00	*0.05*
Desulforhopalus	0.00 ± 0.00	0.01 ± 0.01	n.s.	*Deinococcus*	0.00 ± 0.00	0.01 ± 0.01	n.a.
Methylomonas	0.00 ± 0.00	0.01 ± 0.00	n.s.	*Ehrlichia*	0.00 ± 0.00	0.01 ± 0.00	n.s.
Vibrio	0.00 ± 0.00	0.45± 0.45	n.s.	*Ensifer*	0.00 ± 0.00	0.01 ± 0.00	n.s.
Aminobacter	0.00 ± 0.00	0.07 ± 0.02	*0.05*	*Fretibacterium*	0.00 ± 0.00	0.01 ± 0.00	n.s.
Diaphorobacter	0.00 ± 0.00	0.01 ± 0.00	n.s.	*Frigoribacterium*	0.00 ± 0.00	0.01 ± 0.01	n.s.
Macellibacteroides	0.00 ± 0.00	0.12 ± 0.07	n.s.	*Geosporobacter*	0.00 ± 0.00	0.01 ± 0.01	n.s.
Stigmatella	0.00 ± 0.00	0.01 ± 0.00	n.s.	*Lyticum*	0.00 ± 0.00	0.12 ± 0.12	n.s.
Sulfuricurvum	0.00 ± 0.00	0.05 ± 0.01	*0.05*	*Ohtaekwangia*	0.00 ± 0.00	0.01 ± 0.00	n.s.
Macromonas	0.00 ± 0.00	0.23 ± 0.17	n.s.	*Plasticicumulans*	0.00 ± 0.00	0.01 ± 0.00	n.s.
Xenophilus	0.00 ± 0.00	0.01 ± 0.00	n.s.	*Portibacter*	0.00 ± 0.00	0.03 ± 0.01	*0.05*
Marinithermus	0.00 ± 0.00	0.01 ± 0.00	n.s.	*Pusillimonas*	0.00 ± 0.00	0.11 ± 0.05	n.s.
Alicycliphilus	0.00 ± 0.00	0.02 ± 0.01	n.s.	*Simiduia*	0.00 ± 0.00	0.01 ± 0.01	n.s.
Sunxiuqinia	0.00 ± 0.00	0.01 ± 0.00	n.a.	*Subdoligranulum*	0.00 ± 0.00	0.01 ± 0.01	n.s.
Janthinobacterium	0.00 ± 0.00	0.01 ± 0.00	n.a.	*Thermanaerothrix*	0.00 ± 0.00	0.01 ± 0.00	n.s.
Defluviimonas	0.00 ± 0.00	0.01 ± 0.00	n.a.	*Thiocapsa*	0.00 ± 0.00	0.01 ± 0.01	n.s.

Taxa illustrated in Fig. 1 or with relative abundance below 0.01% are not herein presented. Data is presented as Mean ± SE (n=3). [1], n.s.: not significant; [2], n.a.: not applicable.
